# Editorial: Advances in Molecular Cytopathology

**DOI:** 10.3389/fmed.2022.851949

**Published:** 2022-02-11

**Authors:** Elena Vigliar, Maria D. Lozano, Sinchita Roy-Chowdhuri

**Affiliations:** ^1^Department of Public Health, University of Naples Federico II, Naples, Italy; ^2^Department of Pathology, Clinica University of Navarra, Pamplona, Spain; ^3^Department of Pathology, Anatomy and Physiology, School of Medicine, University of Navarra, Pamplona, Spain; ^4^Department of Pathology, University of Texas MD Anderson Cancer Center, Houston, TX, United States

**Keywords:** molecular cytopathology, next generation sequencing, epigenetic, digital pathology, predictive immunohistochemistry

In the last decade, molecular cytopathology has emerged as a rapidly evolving field of diagnostic and predictive pathology (1), with an increasing number of molecular tests performed with a high degree of versatility on a range of cytological preparations. In fact, from a methodological standpoint, the ongoing development of molecular cytopathology is closely related to the high quality of nucleic acids extracted from cytological samples, and to the rapid advances of highly sensitive and multiplexed molecular assays with minimal nucleic acid requirements. Thus, the role of modern cytopathologists includes, in addition to the morphological evaluation, the adequacy assessment of cytological samples for molecular testing, and the triage and judicious handling of specimens to preserve as much as possible for sample informative integrity.

From a clinical standpoint, cytology is a first-line diagnostic procedure in many neoplastic and non-neoplastic settings. In advanced-stage cancer patients, cytological samples may be the only material available for both diagnosis and molecular biomarker testing to select patients for targeted therapies (2). Interestingly, most contributions to this Research Topic focused on non-small cell lung cancer (NSCLC) setting, in which the molecular assessment of several biomarkers is recommended. In this setting, next generation sequencing (NGS) technology has the advantage of allowing simultaneous detection of predictive biomarkers, even in low-input DNA/RNA specimens, as may often be seen in cytological ones. Here, the review by Pisapia et al. provides an overview of the role of the modern cytopathologist in the morpho-molecular diagnosis of advanced stage NSCLC, focusing on the adoption of currently available NGS assays in different cytopreparations (cell blocks, direct smears, and liquid-based cytology).

RNA and DNA based NGS approaches are also discussed by Schmitt et al., along with immunocytochemistry (ICC), fluorescent *in-situ* hybridization (FISH), reverse transcription-polymerase chain reaction (RT-PCR), as testing method for gene fusion detection; advantages and disadvantages of each assay and specific requirements for the application to routine cytological material are reviewed.

Maximizing cytological material for molecular testing is definitely a relevant goal for modern cytopathologists; in this view, several studies have investigated the possibility of using supernatants for molecular analysis (3). Data from the original research by Patel et al. confirmed that pleural effusion supernatants from patients with metastatic NSCLC represents a robust source of DNA for NGS. Moreover, analytical and pre-analytical challenges in the utilization of cell-free DNA (cfDNA) extracted from pleural effusion supernatant are detailed. In particular, optimization of specimen storage and collection, determination of specimen cellularity, and nucleic acid quantity and quality are identified as challenges in the preanalytic phase; on the other hand, challenges in the analytic phase are represented by selection of a sequencing methodology with appropriate depth, targets, and interpretation of results.

Besides targeted therapy, over the past decade immunotherapy has emerged as one of the most promising cancer treatments (4) and the expression of programmed death ligand-1 (PD-L1), evaluated by immunohistochemistry (IHC), is the main predictive biomarker available for PD-L1/PD1 immunotherapy. As PD-L1 assays have been developed and validated on formalin-fixed paraffin embedded (FFPE) histological specimens, a growing body of research is being dedicated to evaluate the feasibility of PD-L1 IHC assessment also on cytological samples; here, Tejerina et al. summarizes the knowledge of the role of cytopathology in the analysis of PD-L1, addressing important issues such as pre-analytical phase, cyto-histological correlation, and inter-observer agreement.

In addition to the predictive response to tumor targeted treatments, molecular profiling of cytological samples is also currently useful in diagnostics to stratify atypical and undetermined cytology diagnoses into low and high-malignancy risk categories. Moreover, advances in the epigenetic field supporting the differentiation between benign, borderline, and malignant lesions, as well as their role as prognostic factors in malignant neoplasms, are increasingly acknowledged (5). Recently, thyroid also started to be studied by various epigenetic techniques, leading to a growing need to establish the applicability of epigenetic-based methods to FNA material. The review by Canberk et al. covers the main epigenetic categories—DNA methylation, histone modification, and RNA-silencing—and their translational potential on thyroid cytology specimens.

Finally, a new challenge for modern cytopathologists addressed in this Research Topic is represented by the application of digital imaging, artificial intelligence (AI), and multiplex modalities to cytological material (6). Lau et al. reviewed the current state of deep machine learning and AI, that have potential applications to cytology specimens, including exfoliative samples (such as cervical samples, urine, and body cavity fluid samples), fine needle aspiration (FNA), or liquid biopsy samples (circulating tumor cells, CTC); moreover, the authors provided an overview of recent multiplex systems, such as antibody barcoding with cleavable DNA (ABCD), single cell analysis for tumor phenotyping (SCANT), fast analytical screening technique-fine needle aspiration (FAST-FNA), and portable fluorescence-based image cytometry analyzer (CytoPAN), that have shown great promise in their ability to automatically analyze several biomarkers concurrently with high sensitivity, even in paucicellular samples, lending themselves well as tools in FNA.

In conclusion, to this day, cytological samples are still underutilized to select oncological patients for targeted treatments and we argue that a continuous educational effort and a timely update in both clinical and methodological molecular cytopathology aspects are required to better exploit the role of this approach in molecular medicine. All authors contributing to this Research Topic provided an overview of the current state of the art in molecular cytopathology and allowed to convey to the practicing cytopathologist an update on novel testing methodologies and further clarify cytological sample requirements, improving patient management, and treatment.

## Author Contributions

EV, ML, and SR-C equally contributed in conceptualization, writing—review, and editing. All authors contributed to the article and approved the submitted version.

## Conflict of Interest

The authors declare that the research was conducted in the absence of any commercial or financial relationships that could be construed as a potential conflict of interest.

## Publisher's Note

All claims expressed in this article are solely those of the authors and do not necessarily represent those of their affiliated organizations, or those of the publisher, the editors and the reviewers. Any product that may be evaluated in this article, or claim that may be made by its manufacturer, is not guaranteed or endorsed by the publisher.
